# Transcriptome Analysis Reveals the Molecular Mechanism Involved in Carotenoid Absorption and Metabolism in the Ridgetail White Prawn *Exopalaemon carinicauda*

**DOI:** 10.3390/ani15091314

**Published:** 2025-05-01

**Authors:** Yumin Han, Yang Yu, Chengsong Zhang, Shihao Li, Jianbo Yuan, Fuhua Li

**Affiliations:** 1 State Key Laboratory of Breeding Biotechnology and Sustainable Aquaculture, Institute of Oceanology, Chinese Academy of Sciences, Qingdao 266000, China; hanyumin1120@163.com (Y.H.); chszhang@qdio.ac.cn (C.Z.); lishihao@qdio.ac.cn (S.L.); yuanjb@qdio.ac.cn (J.Y.); 2 College of Earth Science, University of Chinese Academy of Sciences, Beijing 100049, China; 3 Laboratory for Marine Biology and Biotechnology, Qingdao Marine Science and Technology Center, Qingdao 266071, China

**Keywords:** astaxanthin supplementation, *Exopalaemon carinicauda*, RNA-seq

## Abstract

Astaxanthin plays a vital role in pigmentation, immune function, reproduction, and antioxidation in aquatic species. To clarify the molecular mechanism of astaxanthin utilization in *Exopalaemon carinicauda* (*E. carinicauda*), we conducted a comparative transcriptome analysis on the intestine, hepatopancreas, and muscle of *E. carinicauda*, fed with an astaxanthin diet and a normal diet. A total of 144 differentially expressed genes (DEGs) were identified in three tissues between the two groups. Genes related to absorption and transport, such as *LDLR* and the vitellogenin receptor, were upregulated in the intestine after astaxanthin supplementation, while the ileal sodium/bile acid cotransporter-like gene was downregulated. In the hepatopancreas, genes involved in lipid storage and degradation were significantly altered at the transcriptional level, including Kruppel 1-like, *ACSBG2*, δ(7)-sterol 5(6)-desaturase-like, and *PNLIPRP2*. In the muscle, the expression of the *FABP* gene was significantly upregulated, while several actin and troponin genes were significantly downregulated. Furthermore, GSEA analysis on the transcriptomes of three tissues revealed that astaxanthin supplementation influenced the expression of genes related to antioxidation and growth, indicating that astaxanthin may have a positive impact on the growth, development, and resistance of organisms. The data from this research provide valuable insights into elucidating the molecular mechanisms underlying astaxanthin absorption and metabolism and also offer guidance for the application of astaxanthin in the aquaculture of economically important crustaceans.

## Simple Summary

This study compared the gene expression in the intestine, hepatopancreas, and muscle of the ridgetail white prawn, *Exopalaemon carinicauda*, fed with or without astaxanthin. The key genes and processes involved in astaxanthin absorption, transportation, and metabolization in shrimp were identified. These results elucidate the molecular mechanisms of carotenoid metabolism in shrimp, offering a reference for future aquaculture nutrition and genetic breeding research and applications.

## 1. Introduction

Astaxanthin (AST), a natural orange-reddish carotenoid, is primarily derived from microalgae, yeast, and certain bacteria. Astaxanthin has been shown to have superior antioxidative activity and the highest oxygen radical absorbance capacity in comparison with other carotenoids [1]. It is an essential metabolic resource for many aquatic organisms, including crustaceans and fish. Astaxanthin plays a crucial role in improving pigmentation, immune function, reproduction, and antioxidation in aquatic species [2]. In crustaceans, the astaxanthin content could affect body coloration, which is a key quality indicator in aquaculture. Additionally, natural AST supplementation offers a safe alternative to antibiotics, addressing concerns over residual drugs in farmed crustaceans and environmental stress. Its role in improving growth, survival, and sustainable aquaculture has spurred increasing interest, and AST supplementation is expected to be a feasible pathway to the sustainable development of aquaculture. However, most animals cannot synthesize carotenoids de novo, and they highly depend on dietary intake or the metabolic conversion of precursors to accumulate astaxanthin in their bodies [3,4].

The absorption, metabolism, transport, and deposition of astaxanthin in shrimp and other crustaceans involve a series of complex processes [5]. The astaxanthin released from dietary sources is absorbed by intestinal epithelial cells, transported through the lymphatic and circulatory systems, and ultimately deposited in various tissues, including the epidermis, muscles, and hepatopancreas [5,6,7]. During these processes, free astaxanthin can undergo esterification or bind with specific proteins, such as crustacyanin (CRN), facilitating its transport and storage within the organism [8]. Despite considerable advances in understanding these mechanisms, key aspects, such as the regulatory pathways governing astaxanthin uptake and deposition, remain insufficiently clarified in crustaceans.

Dietary modification has been widely used as a strategy to improve carotenoid accumulation in aquaculture. For instance, supplementation with lysophosphatidylcholine in *Penaeus vannamei* significantly enhanced astaxanthin deposition in muscle tissues and modulated lipid metabolism by influencing cholesterol transport and apolipoprotein-rich lipoprotein pathways [9]. In *Eriocheir sinensis*, combining docosahexaenoic acid (DHA) with astaxanthin improved carotenoid accumulation in the hepatopancreas and gonads [10]. Additionally, members of the cytochrome P450 family have been identified as potential genes involved in astaxanthin biosynthesis and regulation in *Halocaridina rubra* [11]. These findings collectively highlight the substantial influence of both dietary components and genetic factors on astaxanthin metabolism in crustaceans, underscoring the need for further investigation into these interrelated mechanisms.

Crustaceans offer an attractive model for exploring the metabolism of astaxanthin due to their economic importance and unique biological characteristics, such as their ability to deposit astaxanthin in various forms (e.g., free, monoester, or diester), specifically in tissues like the cuticle and epidermis [12]. For example, dietary astaxanthin has been observed to enhance the antioxidant capacity of *E. carinicauda* by regulating amino acid and lipid metabolism [13]. Moreover, proteins such as crustacyanin, apolipoprotein D (ApoD), and cuticle proteins are thought to play key roles in the transport and storage of astaxanthin in shrimp tissues [14]. Nonetheless, many of the molecular pathways regulating these processes, including transcription factors and enzymes involved in lipid metabolism, require further characterization.

*E. carinicauda* is a commercially valuable decapod crustacean in East Asian aquaculture due to its premium meat quality, rapid growth rate, and resilience to a wide range of environmental conditions [15]. It represents a key species for coastal aquaculture industries, contributing significantly to economies and food security. *E*. *carinicauda* is mainly distributed in the shallow, low-salinity waters along the eastern coastal areas of China and the western coast of the Korean Peninsula. It generally inhabits inshore shallow marine areas, inshore estuaries, and brackish water regions, and can also live in freshwater after acclimation. With its transparent body and short generation cycles, *E. carinicauda* serves as an ideal model for studying the accumulation mechanism of astaxanthin in crustaceans. In the present study, comparative transcriptomic analyses were conducted on the intestines, hepatopancreas, and muscles of *E. carinicauda* with and without dietary astaxanthin supplementation. The objective was to identify critical genes and pathways involved in astaxanthin absorption, transport, and deposition, as well as to explore the immune and antioxidative responses elicited by astaxanthin. These results provide a comprehensive understanding of the molecular mechanisms governing carotenoid metabolism in shrimp, serving as a reference for further studies and practical advancements in aquaculture nutrition and genetic breeding.

## 2. Materials and Methods

### 2.1. Experimental Animals and Astaxanthin Feeding Experiment

Healthy prawns cultured in the laboratory were used for the present study. The average body weight of the prawns was 1.94 ± 0.32 g. They were randomly divided into the experimental group and the control group. Before the experiment, both groups were fed a conventional diet without astaxanthin for two weeks to standardize initial conditions. During the experimental phase, both groups were maintained under similar conditions, including water quality parameters, container specifications, stocking density, and other relevant aquaculture settings, to ensure consistency. Prawns were fed with experimental diets three times daily (7:00, 15:00, 22:00), and the feeding quantity was adjusted to maintain apparent satiation.

The experimental group (WAST group) received a diet supplemented with astaxanthin at a concentration of 400 ppm for two weeks, while the control group (W group) continued to be fed on a standard diet without astaxanthin during the same period. The composition of the experimental diets was the same as previously reported [16]. The diet was formulated by first thoroughly premixing microalgae powder with 5% Ax (DeHe Biotech., Shenzhen, China) and raw egg whites, followed by adding the basal diet and mixing uniformly until well-combined. Wastes, including feces and food residues of experimental prawns, were removed every morning by siphon, and 80% of the water was changed daily. The feed trial lasted for 14 days. During the feed trial, water quality parameters were maintained as follows: temperature, 27 ± 1 °C; salinity, 30 ± 1 PSU; dissolved oxygen, 7.4 ± 0.3 mg L^−1^; pH value, 8.2 ± 0.1; and ammonia–nitrogen, 0.03 ± 0.01 mg L^−1^.

### 2.2. Sample Collection

After the feeding experiments, animals were fasted for 24 h to allow intestinal clearance. *E. carinicauda* were humanely euthanized by rapidly destroying the central nervous system with surgical scissors to ensure immediate death. Subsequently, dissection was performed, and tissues of the muscle, hepatopancreas, and intestine were collected. The collected samples were immediately frozen in liquid nitrogen and then stored at −80 °C for long-term preservation. Three biological replicates were prepared for each tissue type, and each sample was an equal mix of RNA from three experimental animals to ensure experimental reproducibility and reliability. These samples were subsequently used for transcriptome sequencing.

### 2.3. RNA Isolation and cDNA Synthesis

Total RNA was extracted from the samples by RNAiso (TaKaRa, Kyoto, Japan) according to the manufacturer’s instructions. The quality of the extracted RNA was examined by 1% agarose gel electrophoresis, and its concentration was detected using the NanoDrop 2000 spectrophotometer (Thermo Fisher Scientific Inc., Waltham, MA, USA). Quality-qualified samples were used for RNA-seq analysis.

To validate gene expression through real-time PCR, cDNA was synthesized using the PrimeScript RT Kit (TaKaRa, Kyoto, Japan), following the manufacturer’s protocol. Approximately 1 μg of total RNA from each group was used to synthesize complementary DNA (cDNA). Genomic DNA (gDNA) was first removed using the gDNA Eraser provided in the kit. Subsequently, first-strand cDNA was synthesized using the PrimeScript RT enzyme and random primers, which were later used for RT-qPCR validation.

### 2.4. Library Construction and Transcriptome Sequencing

The mRNA was enriched by Oligo (dT) beads, then fragmented by fragmentation buffer, and reverse transcribed into cDNA using random primers. Second-strand cDNA was synthesized by DNA polymerase I, RNase H, dNTP, and buffer. The purified double-stranded cDNA was subjected to end repair, A-tailing, and ligation of sequencing adapters. Subsequently, AMPure XP beads were employed to screen for cDNA fragments. Thereafter, PCR amplification was conducted, followed by a second purification of the PCR products using AMPure XP beads (Beckman Coulter, Brea, CA, USA). Ultimately, sequencing was carried out using the Illumina Novaseq 6000 (Illumina, Albany, NY, New York).

### 2.5. Quality Control, Assembly, and Annotation

Quality control was conducted using fastp to remove adapter sequences and filter out reads containing more than 10% unknown nucleotides or low-quality reads (*Q* ≤ 10). The high-quality reads were mapped to a ribosomal RNA (rRNA) sequence to remove the residual rRNA reads and obtain clean reads. De novo transcriptome assembly of the clean reads was performed using the Trinity program to reconstruct unigenes effectively. For functional annotation analysis, the assembled unigenes were aligned to databases including the National Center for Biotechnology Information non-redundant protein (Nr) database (http://www.ncbi.nlm.nih.gov, accessed on 2 April 2022), the Swiss-Prot protein database (http://www.expasy.ch/sprot, accessed on 2 April 2022), the Kyoto Encyclopedia of Genes and Genomes (KEGG) database (http://www.genome.jp/kegg, accessed on 2 April 2022), and the Cluster of Orthologous Groups (COG/KOG) database (http://www.ncbi.nlm.nih.gov/COG, accessed on 2 April 2022) by blastx (*E* < 10^−5^). Protein functional annotations could then be obtained according to the best alignment results.

### 2.6. Differential Expression Analysis, Gene Annotation, and GO and KEGG Enrichment Analysis

Differential expression analysis of RNA was performed by DESeq2 software between the two groups [17]. Based on the results of the differential analysis, genes with a fold change ≥2 and a false discovery rate (FDR) < 0.05 in the two groups were deemed to be significantly differentially expressed genes (DEGs). Subsequently, the DEGs were subjected to an enrichment analysis of GO functions and KEGG pathways with *p* ≤ 0.05 as the threshold. Then, the hypergeometric test was applied to identify the GO terms and pathways that were significantly enriched among the DEGs, thereby determining the primary biochemical metabolic pathways and signal transduction pathways in which the DEGs were involved.

### 2.7. Gene Set Enrichment Analysis

Gene Set Enrichment Analysis (GSEA) was used to analyze significantly enriched genes in a list of genes ranked by their correlation with a phenotype of interest [18]. In this study, GSEA was used to identify genes that were differentially expressed between the control and experimental groups. All the genes were sorted using the signal-to-noise normalization method. Enrichment scores and *p*-values were calculated using default parameters.

### 2.8. Validation of DEGs by Quantitative Real-Time PCR

Five differentially expressed genes (DEGs) were selected for verification by RT-qPCR. According to the reference sequences of the DEGs, Primer3Plus (https://www.primer3plus.com/index.html, accessed on 27 July 2024) was used to design the specific primers. The information of the primers is shown in Table S1. The 18S rRNA gene was used as an internal reference. According to the instructions, the qPCR was performed using the THUNDERBIRDTM SYBR^®^ qPCR Mix Without ROX kit (TOYOBO, JAPAN). The program was set as follows: One cycle was carried out at 95 °C for 1 min, followed by 40 cycles, with each cycle consisting of 15 s at 95 °C, 15 s at the annealing temperature for each pair of primers, and 30 s at 72 °C, and a melting curve step was added to test the specificity of these primers finally. According to the relative quantification method, the gene expression levels were calculated using the formula 2^−ΔΔCT^ [19]. The qPCR results were compared with the transcriptome data to detect the expression of the selected genes.

### 2.9. Statistical Analysis

The statistical analyses of the qPCR results were performed using SPSS (version 20). All data are presented as means ± standard error of the mean (S.E.M., n = 3). One-way analysis of variance was used to test the differences among the treatments. The difference was considered statistically significant at *p* < 0.05.

## 3. Results

### 3.1. Basic Information of the Transcriptome Sequencing

In this study, 18 cDNA libraries were constructed, with details presented in Table S2. After filtering out low-quality reads and adapters, a total of 44,361,880 reads were retained. These clean reads were subsequently utilized for de novo transcriptome assembly. Consequently, 58,256 Unigenes were obtained, with an average length of 995 bp and an N50 length of 1565 bp. A total of 18,211 unigenes were annotated in the databases. Specifically, 17,918 unigenes were annotated in the Nr database, 11,132 in the Swiss-Prot database, 10,040 in the KOG database, and 17,200 in the KEGG database.

### 3.2. Screening of Differentially Expressed Genes and Functional Analysis

**Figure 1:**
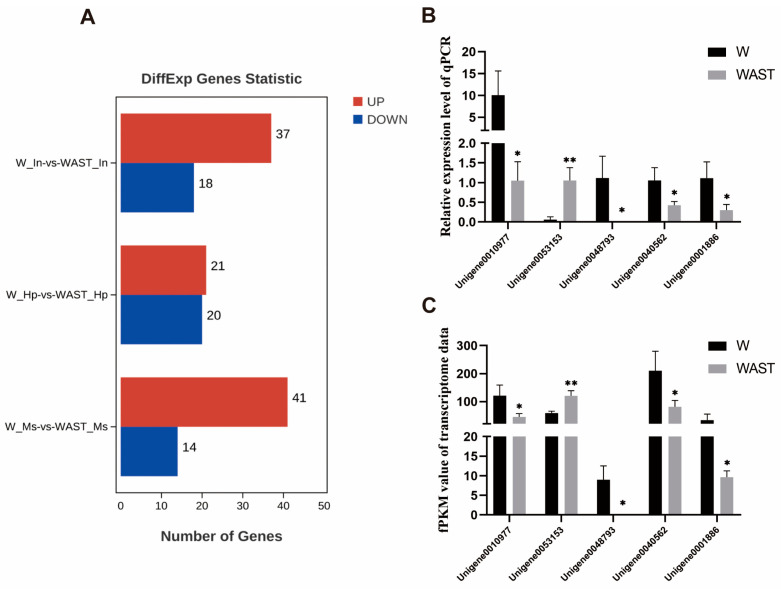
Differentially expressed genes across all groups (**A**). Expression profiles of selected unigenes between the W group and the WAST group. The *x*-axis represents the names of selected unigenes. Columns represent the means of relative expression levels from the qPCR results (**B**) and the FPKM values from the transcriptome results (**C**). Asterisks indicate significant differences in expression levels between the two groups. * indicates *p* < 0.05, and ** indicates *p* < 0.01.

In this study, a total of 144 DEGs were identified between the W group and the WAST group (Table S3). A total of 55, 41, and 55 significant DEGs (FDR < 0.05, |log2(fc)| > 2) were identified in the intestines, hepatopancreas, and muscle tissues, respectively (Figure 1A). Five genes were selected among the DEGs for qRT-PCR validation. These genes include glutathione peroxidase 3, pancreatic lipase-related protein 2-like, C-type lectin 1, organic cation transporter protein-like, and an unknown gene. The qPCR results showed that the expression patterns of the DEGs were consistent with those in the RNA-seq data (Figure 1B,C), indicating that the transcriptome data used in this study and subsequent analyses were reliable.

#### 3.2.1. Intestine Response to AST Supplementation

**Figure 2:**
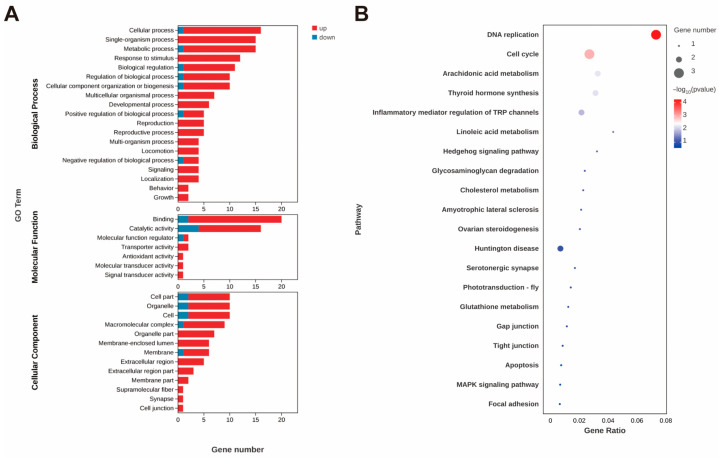
GO annotation of DEGs (**A**) after astaxanthin feeding in the intestines of *E*. *carinicauda*. DEGs with a GO annotation were divided into three major categories: biological process, cellular component, and molecular function. Red columns represented the DEGs with higher expression levels in the experimental group, while blue columns represented the DEGs with lower expression levels in the experimental group. Top 20 enriched KEGG pathways (**B**) in the intestines of *E*. *carinicauda*. The circle size and filled portions represented the number of DEGs in each pathway. The statistical significance was indicated by color, corresponding to *p*-values.

In the intestine, the gene serine/threonine-protein kinase-like, which is involved in protein phosphorylation to initiate various cellular signal transduction pathways, was the most significantly upregulated DEG. The gene exhibiting the most significant downregulation was the ileal sodium/bile acid cotransporter-like (*ASBT*). GO functional annotation of the DEGs in the molecular function category revealed that they were mainly enriched in binding and catalytic activity, in which the genes were primarily upregulated (Figure 2A). Genes potentially involved in the absorption or transportation of astaxanthin, such as low-density lipoprotein receptor-related protein 2-like (*LDLR2*) and vitellogenin receptor (Vg receptor), were upregulated. In contrast, the ileal sodium/bile acid cotransporter-like and vesicle-associated membrane protein 3-like were downregulated.

**Figure 3:**
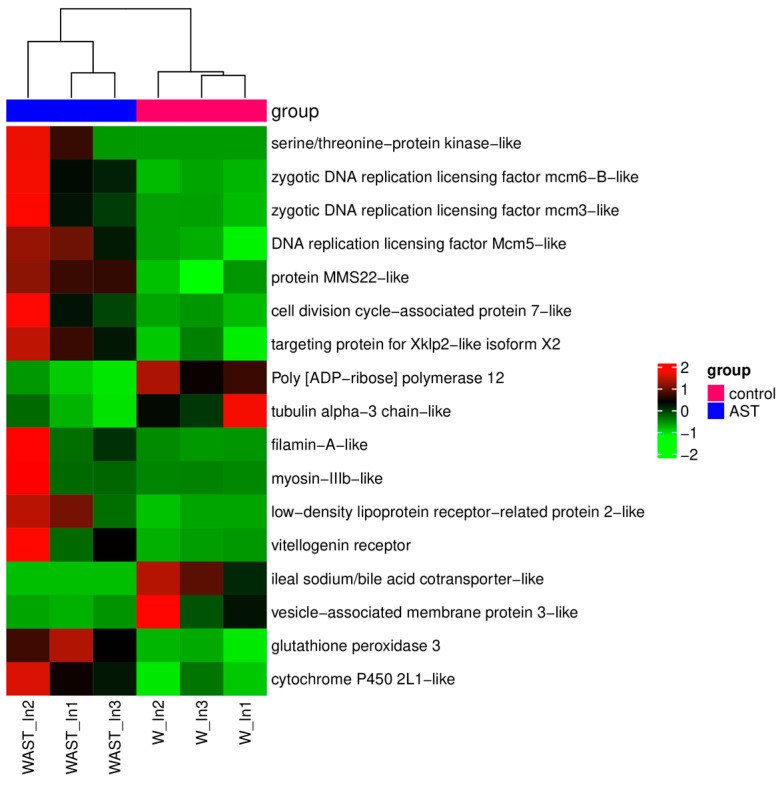
The heatmap of DEGs that are of particular concern after astaxanthin feeding in the intestine. Red and green shadings represent higher and lower relative expression levels, respectively.

In addition, the expression levels of antioxidant and stress-related genes, including glutathione peroxidase 3 (*GPX3*) and cytochrome P450 2L1-like (*CYP2L1*), were significantly upregulated. Moreover, *GPX3* and *CYP2L1* emerged as key genes prominently associated with four enriched KEGG pathways: arachidonic acid metabolism, linoleic acid metabolism, thyroid hormone synthesis, and inflammatory mediator regulation of TRP channels (Figure 2B). In addition, the most significantly enriched KEGG pathways were DNA replication and cell cycle (Figure 2B), where the expression level of the related genes was significantly upregulated (Figure 3).

However, genes such as scavenger receptor class B type I (*SR-BI*), Niemann–Pick C1-like 1 (*NPC1L1*), and ABC transporter families, which are known to be involved in astaxanthin transport, and genes involved in carotenoid metabolism like β-carotene-15,15′-oxygenase (*BCO1*), β-carotene oxygenase 2 (*BCO2*), retinol dehydrogenase, and carotenoid isomerooxygenase, did not show significant differences at transcriptional levels (Table S4).

#### 3.2.2. Hepatopancreas Response to AST Supplementation

**Figure 4:**
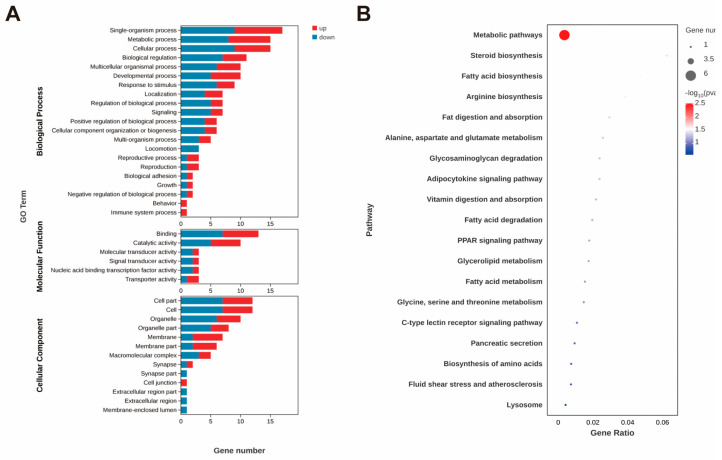
GO annotation of DEGs (**A**) and top 20 enriched KEGG pathways (**B**) after astaxanthin feeding in the hepatopancreas of *E. carinicauda*.

Most of the genes with significantly differentially expressed levels in the hepatopancreas were unannotated. Among these annotated DEGs, the gene with the largest increase was Kruppel 1-like, while the gene with the most significant decrease was facilitated trehalose transporter Tret1-like. Four KEGG pathways, including steroid biosynthesis, fatty acid biosynthesis, fat digestion and absorption, and the adipocytokine signaling pathway, were significantly enriched (Figure 4B). The expression levels of genes related to lipid metabolism, including long-chain fatty acid-CoA ligase ACSBG2-like and δ(7)-sterol 5(6)-desaturase-like, were significantly upregulated, whereas the expression level of pancreatic lipase-related protein 2-like (*PNLIPRP2*) was significantly downregulated.

The genes related to amino acid metabolism were also changed significantly, which include mitochondrial glycine transporter-like, glycine N-methyltransferase-like, and argininosuccinate synthase-like (*ASS*). Furthermore, the expression level of the hyaluronidase-like gene, which may affect intracellular material transport and metabolism by degrading extracellular matrix components, was significantly decreased. In addition, the expression levels of genes that influence growth, development, and molting processes in crustaceans, such as upregulated Mid1-interacting protein, thyroxine 5-deiodinase-like, and downregulated *FTZ-F1-like*, have been identified.

#### 3.2.3. Muscle Response to AST Supplementation

**Figure 5:**
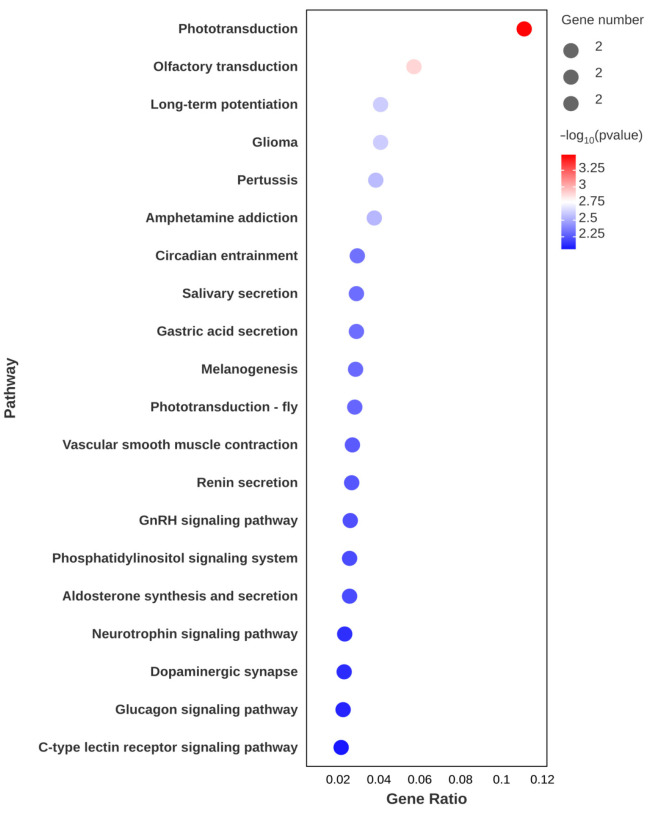
Top 20 enriched KEGG pathways after astaxanthin feeding in the muscle of *E. carinicauda*.

Among the 55 DEGs in muscle, the most significantly upregulated gene was fatty acid binding protein (*FABP*). In contrast, the genes with the most significant downregulation were actin and troponin C-like. The enriched KEGG pathways were mainly related to signal transduction. The DEGs involved in these pathways were mainly two troponin C-like genes with downregulated expression levels (Figure 5). In addition, the expression levels of genes related to growth and development also showed significant upregulation. These included molting-related genes, such as molting fluid carboxypeptidase A precursor, and genes related to extracellular matrix construction, including extensin-1-like. These DEGs identified in muscle were further classified according to their functional annotations, and detailed information is listed in Table S1.

### 3.3. Results of Gene Set Enrichment Analysis

**Figure 6:**
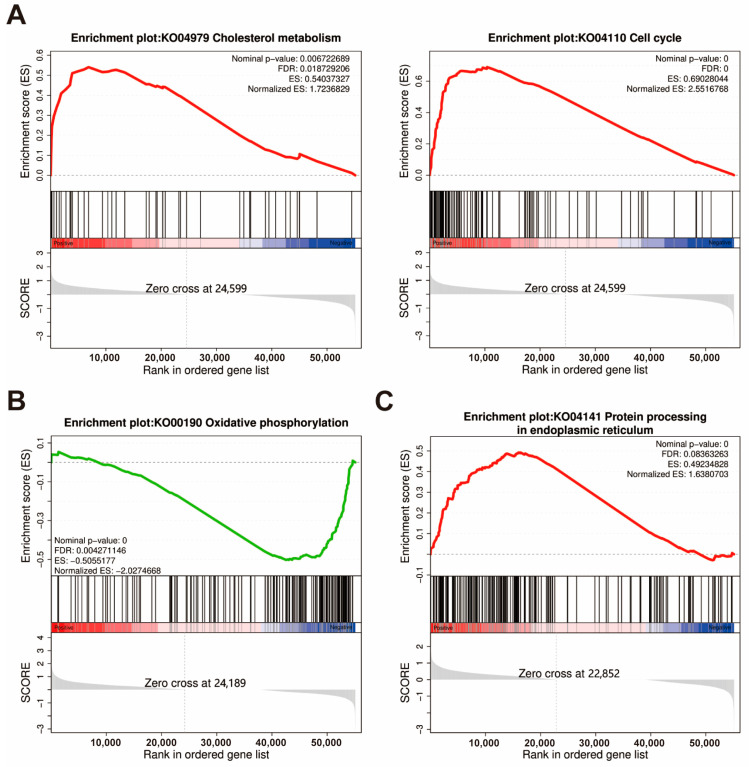
GSEA plots of significantly enriched KEGG pathways are mainly related to astaxanthin accumulation in the intestine (**A**), hepatopancreas (**B**), and muscle (**C**) in the W and WAST groups. Screening criteria for selected KEGG pathways: biological functions of pathways, false discovery rate (FDR) < 0.25, and *p* < 0.05. ES, Enrichment Score.

The results of the GSEA analysis are presented in Table S5, where the key pathway gene enrichments are displayed in Figure 6. In the intestine, the GSEA analysis further revealed that cholesterol metabolism was significantly enriched (NES > 1, q < 0.05), suggesting that the lipid-related pathway may play important roles in influencing the absorption process of astaxanthin. Turning to the hepatopancreas, GSEA analysis also illustrated that oxidative phosphorylation, non-alcoholic fatty liver disease, ribosome biogenesis in eukaryotes, and ribosome pathways were enriched (NES < −2, FDR < 0.05). In the muscle, GSEA analysis revealed that the proteasome (NES > 1, FDR < 0.01) and protein processing in the endoplasmic reticulum (NES > 1, FDR < 0.1), which are involved in protein processing and degradation, were enriched. Pathways potentially involved in astaxanthin metabolism, including oxidative phosphorylation, phagosome, and lysosome, as well as the PPAR signaling pathway and glycerophospholipid metabolism-regulating lipid metabolism and homeostasis (NES > 1, FDR < 0.25), were also among the enriched pathways.

### 3.4. Summary of Metabolic Processes of Astaxanthin in E. carinicauda

**Figure 7:**
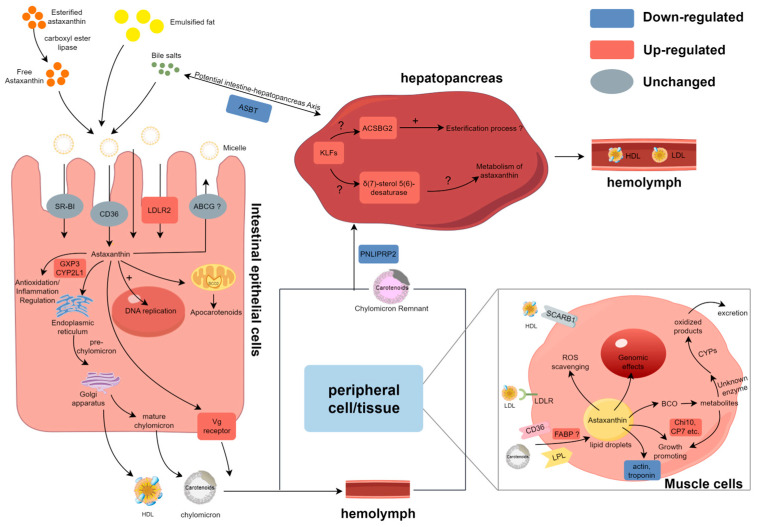
Proposed summary of modulated genes involved in the carotenoid metabolism in these three tissues of the ridgetail white prawn fed astaxanthin. DEGs identified in this study are denoted by red and blue, and gray indicates genes with no significant expression differences. + denotes promotion of this process and ? represents a potential association.

Based on the present data, the general process of astaxanthin absorption and utilization is summarized in Figure 7. In the intestine, the expression of genes involved in the transport of astaxanthin, such as the vitellogenin receptor and potentially *LDLR2* at the apical level, increased when astaxanthin was present in the intestine. The downregulation of the ileal sodium/bile acid cotransporter-like gene expression impaired bile acid reabsorption and influenced mixed micelle formation. Once inside the cell, either astaxanthin crosses the cell and reaches the basolateral membrane, or its metabolization is initiated by *BCO* genes. AST supplementation significantly elevated the expression levels of *CYP2L1* and *GPX3*, facilitating reactive oxygen species (ROS) scavenging and immunomodulation. Additionally, astaxanthin promoted DNA replication processes and cell cycles within intestinal cells. The significantly upregulated serine/threonine-protein kinase-like gene is likely to regulate DNA replication and the cell cycle through the serine and/or threonine phosphorylation of various downstream substrates. Astaxanthin is transported through the hemolymph and circulatory system, eventually reaching peripheral tissues and the hepatopancreas. In the hepatopancreas, the upregulation of the Kruppel 1-like gene likely modulated the activity of long-chain fatty acid-CoA ligase ACSBG2-like and δ(7)-sterol 5(6)-desaturase-like enzymes, potentially influencing astaxanthin esterification and metabolic pathways. Reduced expression of *PNLIPRP2* may compromise hepatopancreatic uptake of astaxanthin-containing lipoproteins. In muscle, the increased expression of fatty acid binding protein (*FABP*) may be associated with the intracellular transport of astaxanthin. The downregulation of actin and troponin genes’ expression under AST supplementation suggests adaptive storage mechanisms or regulatory effects of astaxanthin. Furthermore, AST supplementation altered expression patterns of molting-related genes, implicating its role in growth-associated processes. The expression levels and changes in all key genes responding to AST supplementation are shown in Table 1.

## 4. Discussion

In this study, we performed a comparative transcriptomic analysis of the intestine, hepatopancreas, and muscle of ridgetail white shrimp fed and unfed astaxanthin to explore the molecular mechanisms underlying astaxanthin utilization. While most research on carotenoid feeding in crustaceans has focused on pigmentation, there remains a limited understanding of its broader physiological effects. Notably, much of the available knowledge regarding carotenoid functions originates from studies in fish [12]. Previous studies illustrated that carotenoid metabolism is closely linked to lipids and fatty acids. Our findings identified several differentially expressed genes related to lipid absorption and metabolism. Additionally, we discovered DEGs associated with antioxidant and immune responses, suggesting that astaxanthin might contribute to stress homeostasis via oxidative balance and immune regulation. Furthermore, several DEGs involved in growth and development pathways highlight potential roles for astaxanthin in promoting organismal development. These findings provide new insights into the multifaceted physiological roles of astaxanthin in crustaceans.

### 4.1. Intestinal Absorption of Astaxanthin and Its Impact on the Antioxidant Capacity of the Intestine

Dietary carotenoid esters must first undergo hydrolysis by pancreatic carboxyl ester lipase to release free carotenoids. These free carotenoids, along with other lipids, are emulsified by bile acids to form mixed micelles. These micelles facilitate the efficient transport of carotenoids to the intestinal epithelial surface, where they diffuse and are subsequently absorbed by the intestinal epithelial cells [6]. Higher intestinal bile content, lipase, and dietary lipids promote micellization. Previous studies have shown a 20% increase in blood levels of astaxanthin in Atlantic salmon fed a diet supplemented with taurocholic acid [20]. In this study, the expression of the ileal sodium/bile acid cotransporter-like gene, which is responsible for transporting bile acids from the intestinal lumen back into the intestinal epithelial cells against the concentration gradient, was downregulated after astaxanthin feeding [21]. This may have enhanced the ability to form mixed micelles with astaxanthin in the WAST group. Studies have shown that cholesterol can promote astaxanthin absorption by regulating the retention of membrane proteins and lipoproteins, such as LDLR in the rainbow trout intestine [22]. GSEA analysis revealed that cholesterol metabolism was activated in the WAST group, indicating that after feeding AST, the organism may promote astaxanthin absorption and transport in the gut by influencing lipid transport and cell membrane fluidity.

The mixed micelles then diffuse through the mucus layer of the enterocyte, ready for absorption. Previous studies have shown that some specific membrane proteins and transporters, such as CD36, SR-BI, NPC1, and ABCA1, are involved in carotenoid absorption [23,24]. Although these genes were not altered at the mRNA level in this study, this does not exclude their potential role in astaxanthin absorption. Given the higher expression levels of *NPC1* and some scavenger receptor and ABC transporter family genes in both groups, it is speculated that they may require higher doses of astaxanthin for activation or be activated through non-transcriptional regulatory mechanisms. Notably, low-density lipoprotein receptor-related protein 2-like (*LDLR2*) was upregulated in the WAST group, and we speculate that it may be involved in intestinal astaxanthin uptake and transport, which may be the mechanism of astaxanthin-specific absorption in crustaceans. It has been proposed that astaxanthin is associated with LDL synthesis in enterocytes and transported through potentially LDLR at the apical level in the intestine of Atlantic salmon [25]. Although hepatic uptake of β-carotene has been shown to be mediated by LDLRs [26], this specific mechanism has yet to be confirmed in intestinal tissues. Interestingly, vitellogenin has been implicated as a potential mediator of astaxanthin transport in chum salmon [27]. In line with this, our results demonstrated a significant upregulation of vitellogenin receptor expression in the WAST group, suggesting that this receptor may play a critical role in facilitating the transport of the absorbed astaxanthin–lipoprotein complex to peripheral tissues, such as the hepatopancreas. These findings provide new insights into the molecular pathways underlying astaxanthin transport and deposition in crustaceans.

The absorbed astaxanthin can exert effects on intestinal cells. Astaxanthin effectively increased the integrity of intestinal morphostructure in crucian carp exposed to ethanol [28]. The expression of genes related to DNA replication, repair, and cell cycle (e.g., [ADP-ribose] polymerase 12, zygotic DNA replication licensing factor) was significantly altered in the WAST group. DNA replication and the cell cycle were the most significantly enriched KEGG pathways, and the Hedgehog signaling pathway was activated. These findings suggest that AST supplementation may positively impact intestinal development and repair while simultaneously enhancing the intestinal absorption of astaxanthin. Furthermore, the expression of the mucin-2 gene was significantly upregulated in the WAST group. As a key component of the intestinal mucus layer, mucin-2, synthesized and secreted by goblet cells, forms a protective physical barrier on the intestinal surface. This barrier prevents the invasion of pathogens and harmful substances into intestinal epithelial cells, thereby mitigating epithelial damage caused by mechanical friction [29]. In addition, a significant upregulation of immune- and antioxidant-related genes was observed in the WAST group. Among these, GPX3, the only extracellular glutathione peroxidase (GPX) in the family of oxidoreductases, plays a critical role in catalyzing the detoxification of hydroperoxides and lipid hydroperoxides using reduced glutathione [30]. Similarly, CYP2L1 has been proven essential for detoxification following exposure to heavy metals, such as mercury, in *Procambarus clarkii* [31]. Elevated expression of these genes suggests that astaxanthin, as a natural strong antioxidant, not only enhances the antioxidant capacity of the body but also helps resist inflammation caused by oxidative stress damage.

### 4.2. Hepatopancreas as a Major Site of Astaxanthin Metabolism and Storage

The hepatopancreas is the center of lipid metabolism and the primary storage site for astaxanthin in crustaceans. Kruppel-like factors (KLFs) are a family of zinc-finger transcription factors that are widely involved in critical biological processes, including cell proliferation, differentiation, metabolism, apoptosis, and stress responses. Following AST supplementation, this gene may function as an upstream regulatory factor, responding to oxidative stress signals to enhance the expression of antioxidant-related genes during astaxanthin metabolism, thereby improving cellular antioxidant capacity [32]. Furthermore, this gene is implicated in lipid metabolic processes, not only participating in adipocyte differentiation but also regulating the transcriptional activation of PPARγ at the middle stage of adipogenesis [33]. Based on these facts, we infer that this gene may be involved in the formation of adipocytes and the storage of astaxanthin in the hepatopancreas.

Trehalose is thought to play an essential role in trophic homeostasis and stress tolerance in insects [34]. Trehalose transporter proteins can transport trehalose, depending on intra- and extracellular concentration differences, and regulate its distribution in different tissues. The decreased expression level of the trehalose transporter Tret1-like gene in the WAST group might be attributed to AST supplementation affecting energy metabolism and oxidative stress levels.

Free astaxanthin is usually combined with fatty acids to form astaxanthin esters in the hepatopancreas. ACSBG2 catalyzes the binding of long-chain fatty acids to coenzyme A to generate lipoyl-CoA, which promotes fatty acid activation. Although there is no conclusive evidence, it can be speculated that activated lipoyl-CoA may be involved in astaxanthin esterification, facilitating the transport and utilization of astaxanthin. PNLIPRP2 had a high activity on all phospholipid–bile salt micelles. They can modify the properties of lipid/water interfaces and promote the enzyme–micelle interaction, thus initiating the effective mass transfer between micelles and enzymes during the lipolysis reaction [35]. The expression of *PNLIPRP2* was significantly downregulated in the WAST group, which may reduce the hydrolysis of the phospholipid layer on the surface of celiac particles, while intact celiac particles can be absorbed more efficiently by the hepatopancreas, thereby improving astaxanthin transport efficiency.

As a lipid-soluble compound, the decomposition of astaxanthin involves the participation of multiple genes related to lipid metabolism. We found that the animal δ(7)-sterol 5(6)-desaturase-like is homologous to the gene encoding carotenoid 2,2′-β-hydroxylase from α-Proteobacteria [36]. This animal enzyme usually catalyzes the oxidation of lathosterol to 7-dehydrocholesterol in cholesterol biosynthesis. Although the similarity between the two is not high enough to conclude that the two enzymes have similar functions, they may be involved in their binding to isoprenoid substrates and/or in common oxidation reactions. Further study is needed to determine whether the elevated expression of this gene in the WAST group is associated with the metabolism of astaxanthin.

### 4.3. Muscle Tissue Response to Astaxanthin

FABP exhibits a wide range of ligand specificities and may participate in the intracellular transport of carotenoids. A genetic association study has shown that FABP in the gut is associated with fasting plasma lycopene concentrations, and it has been hypothesized that it binds and carries newly absorbed lycopene in intestinal epithelial cells [37]. In muscle tissue, *FABP* may similarly bind astaxanthin esters, with its upregulated expression potentially aiding in the transport and storage of astaxanthin in muscle cells. Astaxanthin is thought to affect lipid accumulation by modulating PPAR and activating the PPAR pathway [38], and glycerophospholipid metabolism in the WAST group may be relevant. In humans, PPAR-α regulates CD36, which is involved in the transport of fatty acids and AST, and PPAR-γ regulates the synthesis and storage of fatty acids in peripheral tissues [39]. Based on this, it was hypothesized that PPAR may play a role in the uptake and storage of astaxanthin in crustaceans.

Actin is essential in constructing muscle and cellular cytoskeleton. Troponin and actin filaments bind to control muscle contraction in a Ca^2+^-dependent manner. This study observed significant downregulation of actin and troponin C expression in the WAST group, possibly because AST supplementation reduced oxidative stress damage to muscle cells and elevated their stability. Moreover, it was reported that actin microfilaments also play an essential role in the aggregation, dispersion, and maintenance of pigment particles [40]. In *Fenneropenaeus merguiensis*, sarcoplasmic calcium-binding proteins, arginine kinase, and actin promote astaxanthin accumulation [41]. We hypothesized that changes in actin expression in the WAST group may have some connection with adaptation to astaxanthin transportation and storage.

### 4.4. The Impact of Astaxanthin on Gene Expression Regulation Within the Organism

The specific metabolic mechanism of astaxanthin in crustaceans remains unclear and requires further research. GSEA results showed significant enrichment of oxidative phosphorylation, phagosome, and lysosome pathways in muscle, while oxidative phosphorylation was also enriched in the hepatopancreas. Dietary supplementation with astaxanthin helps to maintain mitochondrial function and protects its redox homeostasis. Astaxanthin can prevent mitochondrial dysfunction by permeating and co-localizing within the mitochondria [42]. In addition, astaxanthin regulates redox homeostasis and limits exercise-induced inflammation, thereby protecting mitochondria-rich muscles during exercise [43]. Rainbow trout exhibit reduced carotenoid absorption and accumulation due to mitochondrial dysfunction and impaired oxidative phosphorylation [44]. The activation of the oxidative phosphorylation pathway in the WAST group may be related to astaxanthin metabolism, and the phagosome and lysosome pathways may also be involved in subsequent metabolic processes. Moreover, the activation of the proteins processing in the endoplasmic reticulum and the proteasome pathway are consistent with previous findings [13], suggesting that astaxanthin may be able to promote the clearance of oxidative stress-damaged proteins, but the exact mechanism needs further study.

Additionally, transcriptomic analysis revealed that AST supplementation upregulated genes associated with exoskeleton formation and renewal in muscle tissues, including chitinase 10, molting fluid carboxypeptidase A precursor, and cuticle protein 7, suggesting the importance of astaxanthin in modulating shrimp growth and development. Notably, in the hepatopancreas of the WAST group, significant alterations were observed in the expression of multiple nuclear hormone receptor *FTZ-F1-like* isoforms, which are evolutionarily conserved regulators of cuticle development and molting in insects [45]. This suggests that astaxanthin may exert its effects on crustacean growth and molting processes through the transcriptional regulation of *FTZ-F1*. Furthermore, the marked upregulation of the thyroxine 5-deiodinase-like gene in the hepatopancreas, a key enzyme in thyroid hormone metabolism, likely influences growth-related metabolic pathways, providing additional mechanistic insights into AST-mediated developmental modulation in crustaceans.

## 5. Conclusions

In summary, this study identified several differentially expressed genes between the intestine, hepatopancreas, and muscle of *E. carinicauda* fed either with or without astaxanthin, using transcriptome analysis. Astaxanthin supplementation upregulated genes related to nutrient absorption and lipid metabolism while altering muscle structural gene expression. GSEA revealed enrichment in antioxidation and growth-related pathways, indicating astaxanthin’s potential to enhance growth, development, and stress resistance. The findings of this study offer critical insights into the mechanisms underlying astaxanthin utilization in crustaceans and offer a molecular basis for optimizing its application in sustainable aquaculture to improve crustacean health and production efficiency.

## Figures and Tables

**Table 1:** The expression levels and changes in all key genes responding to AST supplementation.

Gene ID	fpkm_W	fpkm_WAST	log2(fc)	FDR	Description
*Unigene0015436*	4.763	0.001	−12.218	0.00050	ileal sodium/bile acid cotransporter-like
*Unigene0053153*	58.360	120.623	1.047	0.00075	glutathione peroxidase 3
*Unigene0057281*	7.047	17.680	1.327	0.02856	cytochrome P450 2L1-like
*Unigene0058216*	0.193	1.190	2.622	0.01725	vitellogenin receptor
*Unigene0003119*	0.283	1.393	2.298	0.00390	low-density lipoprotein receptor-related protein 2-like
*Unigene0009296*	0.047	24.990	9.065	0.02805	mucin-22-like
*Unigene0004856*	8.497	21.160	1.316	0.00105	zygotic DNA replication licensing factor mcm6-B-like
*Unigene0056003*	6.413	15.893	1.309	0.01734	zygotic DNA replication licensing factor mcm3-like
*Unigene0008818*	10.453	22.793	1.124	0.00361	DNA replication licensing factor Mcm5-like
*Unigene0057764*	6.207	2.633	−1.237	0.00013	Poly [ADP-ribose] polymerase 12
*Unigene0031882*	1.897	17.117	3.174	0.03692	Krueppel 1-like protein
*Unigene0002302*	17.290	47.237	1.450	0.00163	long-chain-fatty-acid-CoA ligase ACSBG2-like
*Unigene0029001*	27.190	60.677	1.158	0.03692	delta(7)-sterol 5(6)-desaturase-like
*Unigene0010977*	121.277	44.893	−1.434	0.00004	pancreatic lipase-related protein 2-like
*Unigene0048711*	0.001	4.410	12.107	0.00617	fatty acid-binding protein
*Unigene0001951*	0.047	7.027	7.234	0.44870	chitinase 10 isoform X4
*Unigene0003091*	0.0433	3.207	6.209	0.00136	molting fluid carboxypeptidase A precursor
*Unigene0001284*	166.973	48.970	−1.770	0.00166	cardiac muscle actin
*Unigene0000217*	7.707	0.633	−3.605	0.00366	actin, muscle-like
*Unigene0042797*	11.293	0.870	−3.698	0.00410	actin, muscle
*Unigene0050957*	20.147	2.233	−3.173	0.01956	troponin C, isotype gamma-like isoform X1
*Unigene0019959*	4.460	0.240	−4.216	0.03396	troponin C-like

## Data Availability

The data presented in this study are available upon request from the corresponding author.

## Supplementary Materials

The following supporting information can be downloaded at: https://www.mdpi.com/article/10.3390/ani15091314/s1. Table S1: Information of primers used for real-time PCR; Table S2: Summary of sequencing and assembly of the transcriptome; Table S3: Information for all the identified DEGs in intestine, hepatopancreas, and muscle of *Exopalaemon carinicauda*; Table S4: Expression of known genes involved in astaxanthin absorption or metabolism in the intestine; Table S5: Enriched KEGG pathways in GSEA analysis.
