# Supplementary material for: Transcriptome Analysis Reveals the Molecular Mechanism Involved in Carotenoid Absorption and Metabolism in the Ridgetail White Prawn Exopalaemon carinicauda
Source: Animals (Basel). 2025 May 1;15(9):1314. doi: 10.3390/ani15091314 (PMC12071124; doi:10.3390/ani15091314)
Supplement: Supplementary file 1 [file animals-15-01314-s001.zip › Table S1 Information of primers used for real-time PCR.pdf]

Table S1. Information of primers used for real-time PCR.

Gene ID	Sequence (5' to 3')	Description
Unigene0053153	CGATTACGCAGAGGAGGACC<br>TTGCCAAACTGGTTGCAAGG	Glutathione peroxidase 3
Unigene0048793	TGACATCCATCCGAGTTGCC<br>AACTCGGATGGATGTCAGCA	NA
Unigene0010977	GACTTCCACCTGATTGGCCA<br>AAAGGGCATCAGAAGGGTCG	Pancreatic lipase-related protein 2-like
Unigene0040562	TGTGAGCAACTACACGGACA<br>GGTGTTCCCATTTTCACGGC	C-type lectin 1
Unigene0001886	GATGAGCCAGGTGGTTTCGA<br>TAAACCGGGAACTCTGCACC	Organic cation transporter protein-like
