# Supplementary material for: Transcriptome Analysis Reveals the Molecular Mechanism Involved in Carotenoid Absorption and Metabolism in the Ridgetail White Prawn Exopalaemon carinicauda
Source: Animals (Basel). 2025 May 1;15(9):1314. doi: 10.3390/ani15091314 (PMC12071124; doi:10.3390/ani15091314)
Supplement: Supplementary file 1 [file animals-15-01314-s001.zip › Table S2 Summary of sequencing and assembly of the transcriptome.pdf]

Table S2. Summary of sequencing and assembly of the transcriptome.

Sample	Raw Reads	Clean Reads	Mapping Ratio	Unigene Number
W_In1	49724248	49609150	42763117 (86.20%)	35734
W_In2	38191800	38107942	33195066 (87.11%)	33055
W_In3	44165788	44065514	38097170 (86.46%)	34688
W_Hp1	44750378	44665868	39395704 (88.20%)	29666
W_Hp2	47953488	47858946	42328175 (88.44%)	31212
W_Hp3	43927432	43850158	38902290 (88.72%)	29895
W_Ms1	44778828	44685076	36293364 (81.22%)	30190
W_Ms2	37673768	37592960	30312141 (80.63%)	25600
W_Ms3	42425030	42334852	34540659 (81.59%)	28713
WAST_In1	51372778	51247396	44671265 (87.17%)	36301
WAST_In2	48834790	48710194	41928464 (86.08%)	36097
WAST_In3	49992524	49869172	43145476 (86.52%)	34923
WAST_Hp1	45484510	45397210	40041190 (88.20%)	31861
WAST_Hp2	37362606	37289616	32761848 (87.86%)	30393
WAST_Hp3	41182696	41100048	36083572 (87.79%)	29331
WAST_Ms1	44184502	440773522	35809306 (81.24%)	29434
WAST_Ms2	39246460	39157072	31826810 (81.28%)	29977
WAST_Ms3	49000098	48895324	39845742 (81.49%)	26880

* Note: Mapping Ratio = Number of Mapped Reads / Number of rRNA-Removed, High-Quality Clean Reads.
