# Supplementary material for: Transcriptome Analysis Reveals the Molecular Mechanism Involved in Carotenoid Absorption and Metabolism in the Ridgetail White Prawn Exopalaemon carinicauda
Source: Animals (Basel). 2025 May 1;15(9):1314. doi: 10.3390/ani15091314 (PMC12071124; doi:10.3390/ani15091314)
Supplement: Supplementary file 1 [file animals-15-01314-s001.zip › Table S4 Expression of genes known to be involved in astaxanthin absorption or metabolism in the intestine.pdf]

Supplementary Table S4. Expression of genes known to be involved in astaxanthin absorption or metabolism in the intestine. These genes with FPKM values less than 1 are considered low-abundance genes and are not listed.

Function	ID	WAST_In_fpkm	W_In_fpkm	FDR	Description
Genes affecting the formation of mixed micelles	Unigene0030205	3.237	2.74	0.997	XP_027214553.1 pancreatic lipase-related protein 2-like
	Unigene0042849	1.303	0.04	0.997	XP_027214553.1 pancreatic lipase-related protein 2-like
	Unigene0011272	7.287	5.55	0.997	XP_027224763.1 pancreatic lipase-related protein 2-like
	Unigene0015482	3.95	2.811	0.997	XP_027224763.1 pancreatic lipase-related protein 2-like
	Unigene0007520	2.04	2.257	0.997	XP_027231664.1 pancreatic lipase-related protein 2-like
Known transporter proteins involved in carotenoid uptake by intestinal epithelial cells	Unigene0002600	2.47	3.00	0.997	ROT78711.1 putative scavenger receptor class B member 1 isoform X2
	Unigene0013579	3.07	3.28	0.997	QCQ82556.1 scavenger receptor class B
	Unigene0039642	6.12	5.11	0.997	AUM57516.1 class B scavenger receptor
	Unigene0020833	27.39	30.14	0.997	ROT83092.1 Niemann-Pick C1 protein
	Unigene0001396	19.167	12.723	0.944	ROT82003.1 putative ATP-binding cassette sub-family A member 12
	Unigene0007936	1.583	0.56	0.586	XP_027224785.1 ATP-binding cassette sub-family

					G member 8-like
	Unigene0007936	1.583	0.56	0.586	XP_027224785.1 ATP-binding cassette sub-family G member 8-like
	Unigene0043387	2.533	5.397	0.692	XP_027236431.1 ABC transporter G family member 20-like
	Unigene0043388	7.217	10.83	0.997	XP_027236431.1 ABC transporter G family member 20-like
	Unigene0028831	139.17	142.47	0.997	XP_027207359.1 ATP-binding cassette sub-family A member 3-like
Genes involved in the transport of carotenoids to the basement membrane of intestinal epithelial cells and chylomicrons secretion	Unigene0049470	7.70	5.99	0.997	ROT79917.1 putative elongation of very long chain fatty acids protein 6-like
	Unigene0049471	95.93	99.30	0.997	ANM86278.1 elongation of very long chain fatty acids protein 6, partial
	Unigene0037659	13.31	14.33	0.997	XP_027233584.1 microsomal triglyceride transfer protein large subunit-like
	Unigene0022168	7339.81	7562.60	0.997	AFD29289.1 fatty acid binding protein 10
	Unigene0044106	201.6	178.39	0.997	AGV76058.1 fatty acid binding protein
	Unigene0048711	6.097	4.54	0.997	AGB13925.1 fatty acid binding protein

	Unigene0037659	13.31	14.33	0.997	XP_027233584.1 microsomal triglyceride transfer protein large subunit-like
	Unigene0006888	11.54	14.32	0.997	QAA06940.1 long-chain fatty acid transport protein 4
	Unigene0056246	4.11	4.55	0.977	XP_027222609.1 long-chain fatty acid transport protein 4-like
Genes involved in carotenoid metabolism in the intestine	Unigene0026416	2.247	0.92	0.736	XP_027229587.1 carotenoid isomerooxygenase-like
	Unigene0004084	0.88	0.943	0.977	XP_027237143.1 beta, beta-carotene 9',10'-oxygenase-like
	Unigene0004144	3.273	3.053	0.977	XP_027214519.1 beta, beta-carotene 9',10'-oxygenase-like
	Unigene0012246	2.903	4.823	0.977	XP_027217427.1 beta,beta-carotene 15,15'-dioxygenase-like isoform X2
	Unigene0015026	24.69	32.727	0.977	XP_027214246.1 beta, beta-carotene 9',10'-oxygenase-like
	Unigene0020799	3.59	3.14	0.977	XP_027214246.1 beta, beta-carotene 9',10'-oxygenase-like
	Unigene0040295	1.383	1.57	0.977	XP_027237143.1 beta, beta-carotene 9',10'-oxygenase-like
	Unigene0039508	1.69	0.553	0.977	QIC55133.1 beta-carotene oxygenase 2
